# Learning the Nonlinear Solitary Wave Solution of the Korteweg–De Vries Equation with Novel Neural Network Algorithm

**DOI:** 10.3390/e25050704

**Published:** 2023-04-24

**Authors:** Ying Wen, Temuer Chaolu

**Affiliations:** 1 College of Information Engineering, Shanghai Maritime University, Shanghai 201306, China; 2 College of Sciences and Arts, Shanghai Maritime University, Shanghai 201306, China; tmchaolu@shmtu.edu.cn

**Keywords:** KdV equation, Lie groups, deep learning, BFGS, partial differential equations

## Abstract

The study of wave-like propagation of information in nonlinear and dispersive media is a complex phenomenon. In this paper, we provide a new approach to studying this phenomenon, paying special attention to the nonlinear solitary wave problem of the Korteweg–De Vries (KdV) equation. Our proposed algorithm is based on the traveling wave transformation of the KdV equation, which reduces the dimensionality of the system, enabling us to obtain a highly accurate solution with fewer data. The proposed algorithm uses a Lie-group-based neural network trained via the Broyden–Fletcher–Goldfarb–Shanno (BFGS) optimization method. Our experimental results demonstrate that the proposed Lie-group-based neural network algorithm can simulate the behavior of the KdV equation with high accuracy while using fewer data. The effectiveness of our method is proved by examples.

## 1. Introduction

Nonlinear science is an interdisciplinary research field that spans a wide range of scientific domains, including life science, mathematical science, spatial science, and geographic science. In recent decades, the study of solitons has become increasingly important and widespread. Solitons are relevant in many fields of science, including fluid mechanics, quantum mechanics, biology, ocean engineering, and more. Therefore, the solution of soliton equations is theoretically and practically critical and has become an important area of theoretical and application-based research. The investigation of soliton equation solution techniques has not halted since the soliton theory was first put forward. Numerous equations have verified several mature solution techniques, including the Painlevé analysis [1], the Backlund transform method [2], the Darboux transform method [3], the inverse scattering transform method [4], the Lie group and Lie algebra method [5], the Hamiltonian structure method [6], etc. There are numerous approaches for finding the exact solution to the soliton problem due to its complexity; however, these approaches cannot be unified.

The ability to answer such scientific computing issues in conjunction with numerical analysis has significantly improved with the development of machine learning [7]. The most well-known area of machine learning research is deep learning. Researchers originally proved a general approximation theorem [8] for neural networks, and deep learning was later used in a variety of fields, including image recognition [9], natural language processing [10], and optimization problems [11]. The neural networks have an advantage over the dimensional catastrophe problem that traditional numerical approaches must deal with because of the increased dimensionality and the linear rise in computational effort. Differential equations and machine learning methods combined have been proven to be advantageous. The combination of differential equations and machine learning methods is beneficial in solving soliton problems. The performance of neural network-based algorithms in predicting soliton solutions has been investigated, and these algorithms have demonstrated promising results in terms of accuracy and the amount of required data. With ongoing developments in machine learning, there is potential for further advancements in the field of soliton equation solutions. Overall, the integration of machine learning and soliton theory is a promising approach that can lead to significant progress in various fields of science.

Recent advancements in deep neural networks have enabled researchers to solve differential and partial differential equations (PDEs) with fewer data points while maintaining high accuracy, making deep learning techniques an increasingly popular alternative to traditional numerical methods. In the beginning, Lee et al. [12] employed Hopfield neural networks to solve ordinary differential equation (ODE) models. Lagaris et al. [13] obtained the trial solution within the error range by continuously optimizing the parameters of the neural network and replacing the solution of the equation with the sum of the initial and boundary value and the neural network function. Methods that require fewer data and produce quick results while maintaining high accuracy are gaining popularity. Chen et al. [14] used neural networks to parameterize the derivatives of the hidden states rather than directly parameterizing the hidden states, auto memory tuning, and adaptive computation to compress the ODEs into the neural network. Raissi et al. [15,16] suggested the novel loss function form to physical priors into the neural network architecture. Differential equations and machine learning techniques are combined in the scientific machine learning that Rackauckas et al. [17] proposed. Continuous convolutional neural networks were employed by Habiba et al. [18] to learn PDE systems. Deep learning in machine learning is increasingly being used as a framework to analyze PDEs, including the common nonlinear wave model KdV equation. Cellular neural networks can imitate KdV behavior, as demonstrated experimentally by Bilotta et al. [19]. To forecast the solutions and parameters of the KdV equation, Fang et al. [20] integrated conservation laws into neural networks. Higher-order nonlinear soliton equations were solved by Cui et al. [21] using deep learning techniques. A two-stage physics-informed neural network (PINN) approach was suggested by Lin et al. [22] to more accurately and generally simulate the local wave solutions of the productive equations. Lin et al. [23] followed up by using the Miura transform and PINN to propose a PINN scheme based on Miura transform to solve the KdV equation. Wu et al. [24] conducted a comprehensive study on the sampling method of PINN sampling and tested its performance in the KdV equation, guiding researchers on the sampling method in subsequent research. Applications of deep learning in analyzing PDEs, including the common nonlinear wave model KdV equation, are gaining popularity. The literature [25] provides a review of the broad application potential of deep neural networks for solving PDEs. This approach is expected to be more widely adopted in future research, facilitating the progress of scientific areas such as physics, biology, and finance.

While neural networks have been used to solve PDE problems, the development of efficient algorithms that utilize minimal resources whilst still effectively addressing the underlying properties of solutions remains an ongoing issue. In this paper, we propose a novel approach that utilizes a Lie-group-based neural network algorithm for solving the KdV equation. Our new method boasts good learning performance, which we affirm by comparing its numerical results with those obtained from the true solution. Specifically, inspired by the unique form of Lie group theory for solving first-order differential equations, we developed a novel method to address PDE problems by constructing a solution consisting of a neural network function and a Lie-group-based solution. In our approach, the sum of these two parts approximates the solution of the differential equation. To effectively apply this approach to PDE problems, we first convert the PDEs into an ODEs. We observed that constructing the solution in this manner eliminates the need to increase the initial value item in the loss function whilst still fully satisfying the initial value. Moreover, using only a small number of neural network parameters can improve fitting ability, all thanks to the Lie-group-based solution. Our proposed approach is highly efficient since the Lie-group-based solution captures the nonlinear characteristics of the KdV equation well before training the neural network. As a result, the cost of the subsequent neural network calculations is reduced. Our approach not only delivers precise predictions but also highlights essential characteristics of the KdV solution such as the constancy of solitary waves over time. By leveraging our method, we can better understand and analyze complex physical phenomena described by the KdV equation, a feat that has remained challenging using other techniques. The ability to capture these key features is crucial in advancing our understanding of nonlinear dynamics and provides a significant boost to the predictive power of our model.

Encouragingly, our investigation revealed that this new method can efficiently and accurately capture complex phenomena in nonlinear waves. We developed all implementations using PyCharm 2021.2.3 and conducted simulations on a Lenovo laptop with a 2.60 GHz 2-core Intel(R) Core(TM) i5-3230M CPU and 8GB memory. The proposed approach could serve as a foundation for future research exploring more general forms of PDEs while reflecting upon the properties of the solutions. The code used in this study is made publicly available to support reproducibility and ease further analyses.

The remainder of this essay is structured as follows. The algorithm presented in this paper and its precise steps are shown in Section 2. The approach is used to solve the KdV equation in Section 3, and this section goes into great depth about how it was accomplished and how accurate the results were. We also study and evaluate our results. Concluding comments and future research work are offered in Section 4.

## 2. The Main Idea of the Lie-Group-Based Neural Network Algorithm

### 2.1. Illustration of the Algorithms

Consider the general form of PDE as follows:

(1) $$u_{t}+N\left(x,u,u_{x},u_{xx},\cdots \right)=0,\;x\in \Omega ,\;t\in \left[0,T\right].$$

The independent variables *x*, *t*, the solution *u* to be solved, and the partial derivatives of *u* with respect to the space variable *x* make up the nonlinear function *N*. The equation is subject to boundary or initial conditions.

The following autonomous system of ODEs is obtained by transforming [26] the PDE:

(2) $$\begin{array}{ccc} & & \frac{du_{i}}{da}=f_{i}\left(u_{1},u_{2},\cdots ,u_{n}\right),\;\;u_{i}\left(0\right)=\alpha_{i}\in R^{1},i=1,2,\cdots ,n. \end{array}$$

where $a\in O\subset R^{1}$ is independent variable *x* or *t* and $u_{i}=u_{i}\left(a\right)$ is *u* in (1) and $f_{i}$ are differential functions of own arguments after the variable *t* or *x* has been eliminated. $\alpha_{i}$ is the initial condition.

From [27], the solution of (2) can be written as Lie group solution $\hat{u}\left(a\right)=e^{aD}\alpha$, where *D* is the differential operator. According to theorem 2, *D* can be split into $D_{1}+D_{2}$, $e^{aD}=e^{aD_{1}}+\sum_{\alpha =1}^{\infty }\sum_{k=\alpha }^{\infty }\frac{a^{k}}{k!}D_{1}^{k-\alpha }D_{2}D^{\alpha -1}$. The first part $e^{aD_{1}}$ is obtained from the equation $\frac{d\bar{u}}{da}=D_{1}\bar{u},\bar{u}\left(0\right)=\alpha$, the second integral calculation should be replaced with the neural network function form aN(a;θ), which offers superior simplicity and ease of computation. The advantage of $\hat{u}\left(a\right)=\bar{u}\left(a\right)+aN\left(a;\theta \right)$ for approximating the solution *u* of the (2) is that the first part can easily capture the nonlinear nature of the equation which can accelerate the convergence of the second part of the neural computation, while the second part uses a simple neural network structure with fewer resources and less memory consumption, and the sum of the two parts can effectively model the behavior of (1).

In our study, we use a fully connected neural network N with one input, one output, *m* units in the hidden layer, and an activation function σ. The outputs of the network N can be written as

(3) $$\begin{array}{ccc} & & N\left(a;\theta \right)=Z_{L}\\ & & =\sigma (W^{\left(L\right)}\cdots \sigma \left(W^{\left(l\right)}\cdots \sigma \left(W^{\left(2\right)}\cdot \sigma \left(W^{\left(1\right)}\cdot a+b^{\left(1\right)}\right)+b^{\left(2\right)}\right)\right)+b^{\left(L\right)}). \end{array}$$

The output of the layer *l* is $Z_{l}=\sigma \left(W^{\left(l\right)}\cdot Z_{l-1}+b^{\left(l\right)}\right)$, ${\left\{a_{\tau }\right\}}_{\tau =1}^{\lambda }$ is the training point in the definition domain, $\theta ={\left\{W^{\left(l\right)},b^{\left(l\right)}\right\}}_{l=1}^{L}$ is the parameters of the neural network, where $W^{\left(l\right)}$ is the weight of layer *l* with respect to layer l−1, $b^{l}$ is the bias of layer *l*, and $w_{jk}^{\left(l\right)}$ is the weight from units *k* in the layer l−1 to units *j* in the layer *l*. By adjusting the parameter θ, we can enhance the approximation of u(a) via the network solution $\hat{u}\left(a\right)$ where σ is a nonlinear activation function $\tanh=\frac{e^{x}-e^{-x}}{e^{x}+e^{-x}}$.

(4) $$\begin{array}{c} W^{\left(l\right)}=\left(\begin{array}{ccc} w_{11}^{l} & \cdots & w_{1m_{k-1}}^{l}\\ \vdots & \ddots & \vdots \\ w_{m_{k}1}^{l} & \cdots & w_{m_{k}m_{k-1}}^{l} \end{array}\right), \end{array}$$

(5) $$\begin{array}{c} b^{\left(l\right)}=\left(\begin{array}{c} b_{1}^{l}\\ b_{2}^{l}\\ \vdots \\ b_{m_{k}}^{l} \end{array}\right).\; \end{array}$$

### 2.2. Details of the Algorithm

The unconstrained optimization process of (2) is measured by the following mean square error equation

(6) $$\begin{array}{c} \begin{array}{c} 𝓛\left(\theta \right)=\frac{1}{\lambda }\left(\sum_{\tau =1}^{\lambda }\sum_{i=1}^{n}{\left({\hat{u}}_{i}^{\prime }\left(a_{\tau }\right)-f_{i}\right)}^{2}\right), \end{array} \end{array}$$

The trial solution $\hat{u}\left(a\right)$ is substituted into (2) so that the loss function (6) of the neural network is minimized at the training points, and the parameter set {W,b} is found using the optimization algorithm. λ is the number of training points and *n* denotes the number of equations. When the number of equations increases, the number of training points can be increased. The method can successfully approximate the solution *u* of (1) when 𝓛(θ) is small enough. In addition to using the mean square error mentioned above to create the loss function, we also used the average root mean square error $L_{RMSE}$ to evaluate the superiority of the method.

(7) $$\begin{array}{c} L_{RMSE}=\frac{1}{2}\sum_{\mu }L_{\mu }\left(\theta \right), \end{array}$$

where $L_{\mu }\left(\theta \right)$ is the mean square error between the trial solution ${\hat{u}}_{i}\left(a\right)$ and the exact solution $u_{i}\left(a\right)$. When the exact solution is not available, the numerical solution $u_{i}\left(a\right)$ is employed, μ is the number of dependent variables, where $L_{1}\left(\theta \right)=\sqrt{\frac{1}{\lambda }\left(\sum_{\tau =1}^{\lambda }{\left(\hat{u}\left(a_{\tau },\theta \right)-u\left(a_{\tau }\right)\right)}^{2}\right)}$.

## 3. Example for Korteweg–De Vries Equation

In this study, we present our novel method for identifying solitons of the KdV equation. The KdV equation represents a fundamental model in mathematical physics and is typically formulated as a PDE given by $u_{t}+6uu_{x}+u_{xxx}=0$. This equation is commonly used to describe water waves and has been extensively studied in the previous literature [28].

To detect the soliton of the equation, we first perform a traveling transformation denoted by ξ=x−vt, thereby enabling us to transform the PDE into an ODE. Specifically, this transformation allows us to rewrite the equation in terms of the new variable ξ as

(8) $$u^{\prime \prime \prime }+6uu^{\prime }-vu^{\prime }=0,$$

with u=u(ξ). We seek the soliton to the (8) with properties $u\left(0\right)=u_{max},u^{\prime }\left(0\right)=0,u^{\prime \prime }\left(0\right)=u_{0}<0$ and u(±∞)=0. Specifically, when ξ=0, the wave value reaches its peak at u(0)=1. In our particular case, we take v=2, $u_{\max}=1$, $u_{0}=-1$ and consider the variable ξ over the interval [−3,3].

This ODE formulation can be solved using our proposed method, which efficiently detects soliton solutions in the equation. We transform the problem (8) to the standard form in our method as

(9) $$\begin{array}{ccc} & & {\dot{u}}_{1}=u_{2},\;\;{\dot{u}}_{2}=u_{3},{\dot{u}}_{3}=2u_{2}-6u_{1}u_{2}, \end{array}$$

with initial values $u_{1}\left(0\right)=1,u_{2}\left(0\right)=0,u_{3}\left(0\right)=-1$ by introducing variables $\left(u_{1},u_{2},u_{3}\right)=\left(u,\dot{u},\ddot{u}\right)$. It corresponds to operator $D=D_{1}+D_{2}$ with a selection $D_{1}=u_{2}\partial_{u_{1}}+u_{3}\partial_{u_{2}}+2u_{2}\partial_{u_{3}}$.

The associated initial value problem yields solutions

$$\begin{array}{ccc} & & {\bar{u}}_{1}\left(\xi \right)=-\frac{1}{2}\left(\cosh(\sqrt{2}\xi )-3\right);{\bar{u}}_{2}\left(\xi \right)=-\frac{1}{\sqrt{2}}\sinh\left(\sqrt{2}\xi \right);\\ & & {\bar{u}}_{3}\left(\xi \right)=-\cosh\left(\sqrt{2}\xi \right). \end{array}$$

Therefore, we have trial solution $\hat{u}=\bar{u}+\xi N\left(\xi ,\theta \right)$. The parameters of the neural network N can be learned by minimizing the mean squared error loss (6)

(10) $$𝓛\left(\xi ,\theta \right)=\frac{1}{\lambda }\left(\sum_{\tau =1}^{\lambda }{\left({\hat{u}}_{1}^{\prime }\left(\xi_{\tau }\right)-f_{1}\right)}^{2}+{\left({\hat{u}}_{2}^{\prime }\left(\xi_{\tau }\right)-f_{2}\right)}^{2}+{\left({\hat{u}}_{3}^{\prime }\left(\xi_{\tau }\right)-f_{3}\right)}^{2}\right),$$

where $f_{1}={\hat{u}}_{2}\left(\xi_{\tau }\right)$, $f_{2}={\hat{u}}_{3}\left(\xi_{\tau }\right)$, $f_{3}=2{\hat{u}}_{2}\left(\xi_{\tau }\right)-6{\hat{u}}_{1}\left(\xi_{\tau }\right){\hat{u}}_{2}\left(\xi_{\tau }\right)$, τ=1,2,⋯,λ.

**Figure 1:**
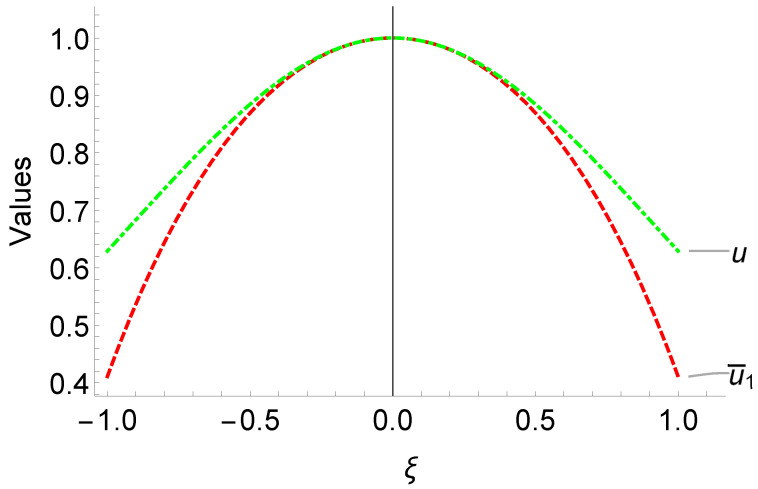
Comparison of the efficiency of the first part ${\bar{u}}_{1}$ of the network solution ${\hat{u}}_{1}$ with the exact solution $u={\sec h}^{2}\left(\xi /\sqrt{2}\right)$ of problem (8).

The comparisons of our solution ${\bar{u}}_{1}$ and exact solution $u={\sec h}^{2}\left(\frac{\xi }{\sqrt{2}}\right)$ to (8) are given in the Figure 1. Our proposed method has been able to accurately approach the true solution in the range of interest [−1,1], indicating the first part of our solution is effective. This not only improves the accuracy of our solution but also speeds up the computation of the second part of our neural network, which ensures rapid convergence.

**Figure 2:**
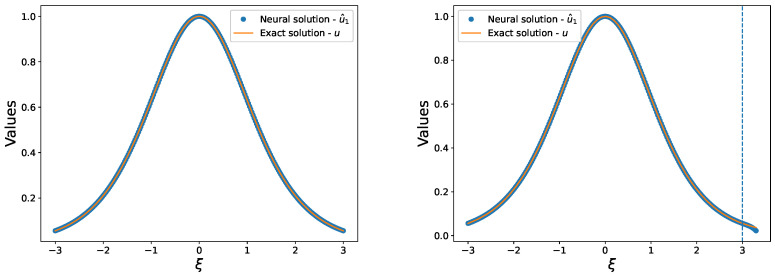
(**Left**): Comparison of our solution $\hat{u}$ with the exact solution $u={\sec h}^{2}\left(\xi /\sqrt{2}\right)$ of problem (8) in the training set. (**Right**): Comparison of our solution $\hat{u}$ with the exact solution $u={\sec h}^{2}\left(\xi /\sqrt{2}\right)$ of problem (8) in the test set.

In this study, we investigate the ability of our proposed method to learn from sparse training data. To accomplish this, we obtained a limited set of 250 training data within the interval [−3,3], which were used to train a feed-forward neural network with a single hidden layer consisting of 30 units. The network solution $\hat{u}$ for the initial value problem (8) was obtained by minimizing the mean square error of (6) using Broyden-Fletcher-Goldfarb-Shanno (BFGS) algorithm [29]. The comparison of the network solution $\hat{u}$ with the exact solution *u* on the training set is presented in the left panel of Figure 2. Our method achieves an accurate approximation of the exact solution *u* even when trained with small amounts of data. The right panel of Figure 2 shows the comparison of the network solution $\hat{u}$ with the exact solution *u* within the test set [−3,3.3]. We observe that the network maintains good generalization ability outside of the interval [−3,3] and continues to provide accurate approximations of the solution in the absence of training points. Our results suggest that the proposed approach is capable of learning from limited training data, which is particularly important in practical scenarios where data acquisition may be difficult or expensive.

**Figure 3:**
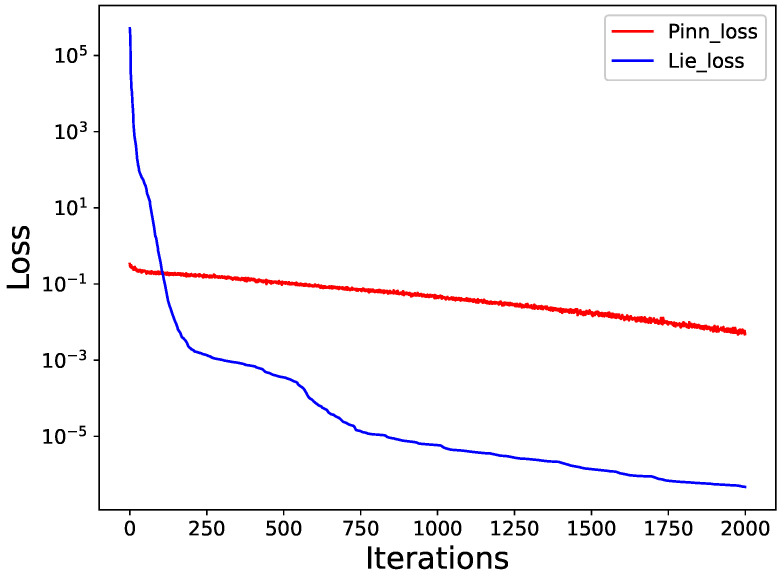
The variation curve of the loss function with the number of iterations for the PINN approach and the Lie-group-based neural network algorithm for problem (8).

The blue line in Figure 3 shows the relationship between the number of iterations during training and the loss function 𝓛(ξ,θ), an error entirely attributed to the Lie group method and the neural network’s ability to approximate *u*. When the number of iterations is around 2000, $𝓛\left(\theta \right)=2.8378\times 10^{-7}$. $L_{RMSE}=7.6167\times 10^{-5}$. To evaluate the effectiveness of our proposed method, we compare it with existing PINN approaches. We conduct experiments using 250 training points in the interval x∈[−1,3],t∈[0,1] and a single hidden layer structure with 30 neurons. The loss function is set to $L=\frac{1}{\lambda }\sum_{\tau =1}^{\lambda }\left(L_{f}+L_{u}\right)$, where $L_{f}={\left({\hat{u}}_{t}+6\hat{u}{\hat{u}}_{x}+{\hat{u}}_{xxx}\right)}^{2}$, $L_{u}={\left(\hat{u}\left(0,x^{\tau }\right)-u\left(0,x^{\tau }\right)\right)}^{2}+{\left(\hat{u}\left(t^{\tau },-1\right)-u\left(t^{\tau },-1\right)\right)}^{2}+{\left(\hat{u}\left(t^{\tau },3\right)-u\left(t^{\tau },3\right)\right)}^{2}.$ The objective is to approximate the exact solution *u* with higher accuracy under the same conditions as our proposed method. We compare the performance of our method with other PINN algorithms by the number of iterations versus the loss value. Our experimental results show that the PINN method produces better loss function values until the number of iterations is 100, and our proposed method outperforms existing methods in terms of convergence speed and accuracy after 100 iterations. As described in Figure 3, the loss function *L* of PINN method reaches $10^{-3}$ after about 2000 iterations.

We investigate the prediction accuracy of several neural network architectures using the same training points to analyze the performance of our proposed method in more detail. We study the loss function 𝓛(θ) for different numbers of hidden layers and different numbers of neurons per layer. Table 1 presents the results of our analysis, demonstrating the impact of varying the architecture of the neural network on prediction accuracy. Here, the training points are fixed to the range [−3,3] of 250 uniformly spaced points. As expected, we observe that as the number of layers and neurons increases, the prediction accuracy systematically improves. This is in line with the general notion that larger and deeper neural networks have greater expressive power and are better equipped to approximate complex functions. It is worth noting that while increasing the number of neurons and layers in a neural network can improve its performance, it also comes at a cost of increased computational complexity and potentially slower training times. Therefore, in practical settings, it is important to balance the trade-off between model complexity and computational efficiency. Our findings suggest that, given sufficient training data, our proposed method can be used to build highly accurate models, but careful consideration must be given to the size of the neural network when implementing it in practical applications.

**Figure 4:**
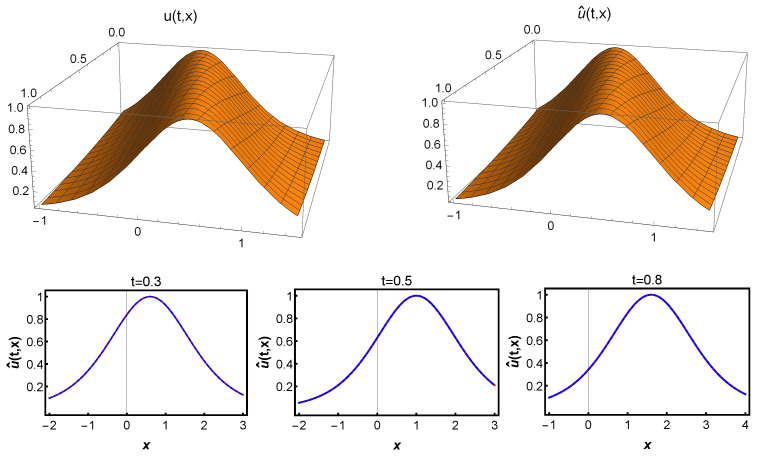
(**Top**): The true solution $u={\sec h}^{2}\left(\left(x-2t\right)/\sqrt{2}\right)$ of the KdV equation is on the left, the predicted solution $\hat{u}\left(t,x\right)$ is on the right. (**Bottom**): Comparison of predicted and exact solutions at time t=0.3, 0.5, and 0.8. (The dashed blue line indicates the exact solution u(t,x), and the solid red line indicates the predicted solution $\hat{u}\left(t,x\right)$).

The results of this experiment are summarized in Figure 4. Specifically, the top left panel shows the true solution u(t,x) of the KdV equation, while the right panel displays the spatiotemporal solution $\hat{u}\left(t,x\right)$ predicted according to the chosen optimal parameter θ. We observe that our approach is highly accurate in approximating the true solution. The bottom panel of Figure 4 gives a more detailed evaluation of the predicted solution $\hat{u}\left(t,x\right)$. For different times t=0.3, 0.5, and 0.8, we compare the exact and predicted solutions in particular at the bottom of Figure 4. Our experimental results demonstrate that our approach can produce highly accurate predictions even for complex spatiotemporal problems such as the KdV equation.

To further investigate the effectiveness of the algorithm in approximating the performance of the true solution of the KdV equation, with the true solution $u=2/3-2\tanh^{2}\left(\xi \right)$ [30], the solitary wave with wave peak u(ξ)=2/3 is sought under the traveling wave transform ξ=x+4t with the initial conditions $v=-4,u_{max}=2/3,u_{0}=-4$ and consider the interval [−3,3] for variable ξ.

**Figure 5:**
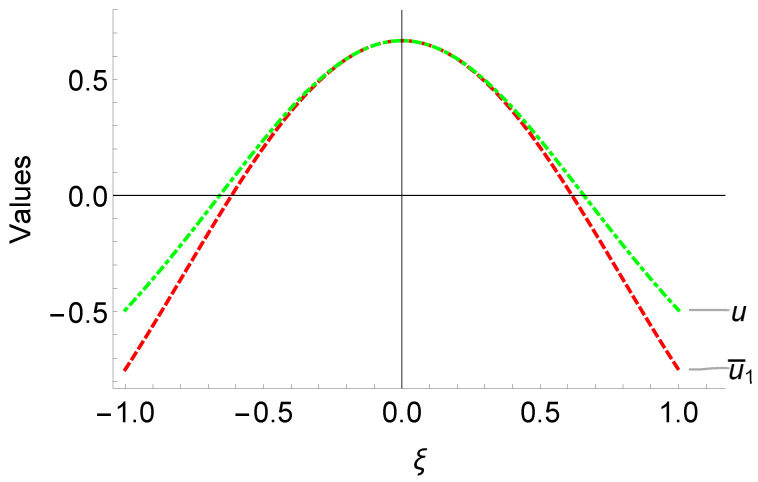
Comparison of the efficiency of the first part ${\bar{u}}_{1}$ of the network solution ${\hat{u}}_{1}$ with the exact solution $u=2/3-2\tanh^{2}\left(\xi \right)$ of problem (8).

The initial value problem $u_{1}\left(0\right)=2/3,u_{2}\left(0\right)=0,u_{3}\left(0\right)=-4$ for problem (9). Choose the operator $D_{1}$ that $u_{2}\partial_{u_{1}}+u_{3}\partial_{u_{2}}-4u_{2}\partial_{u_{3}}$. The associated initial value problem yields solutions, ${\bar{u}}_{1}\left(\xi \right)=-\frac{1}{3}\cos\left(2\xi \right)$, ${\bar{u}}_{2}\left(\xi \right)=-2\sin\left(2\xi \right)$ and ${\bar{u}}_{3}\left(\xi \right)=-4\cos\left(2\xi \right)$. The decomposition part of the trial solution constructed from the Lie group can capture the nonlinear nature of the problem (8), as illustrated by a comparison between *u* and ${\bar{u}}_{1}$ in Figure 5.

**Figure 6:**
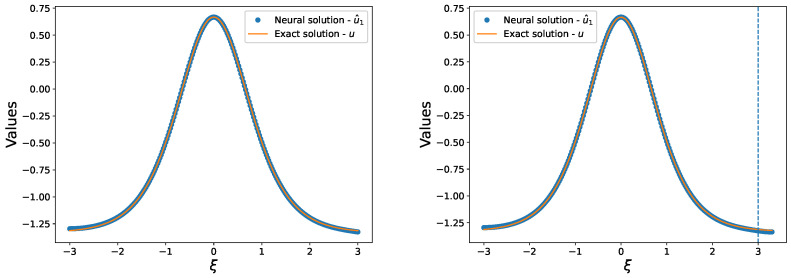
(**Left**): Comparison of our solution $\hat{u}$ with the exact solution $u=2/3-2\tanh^{2}\left(\xi \right)$ of problem (8) in the training set. (**Right**): Comparison of our solution $\hat{u}$ with the exact solution $u=2/3-2\tanh^{2}\left(\xi \right)$ of problem (8) in the test set.

Using the BFGS algorithm, the same network structure with a single hidden layer containing 30 neurons, training data equally spaced at 250 training points in the range [−3,3], and the same test data are learned *u*. The results are presented in Figure 6, where it is evident that our approach yields remarkable accuracy in predicting *u*.

**Figure 7:**
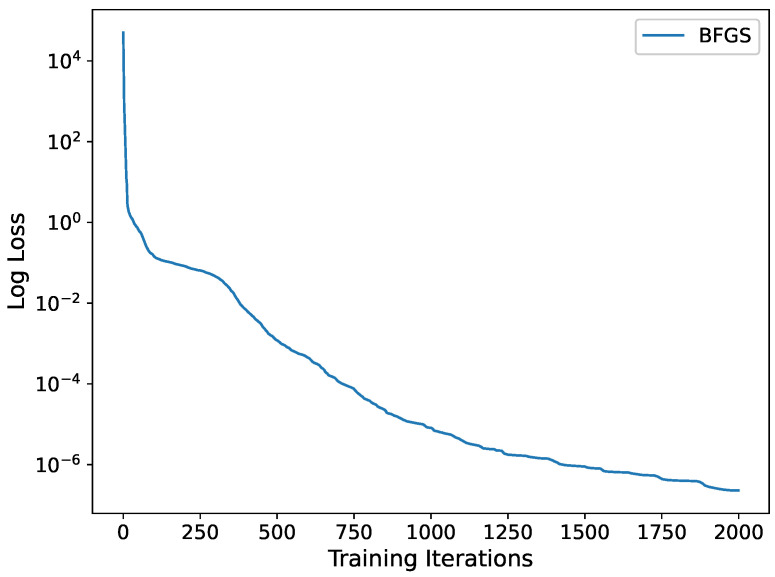
The variation curve of the Loss function with the number of iterations for the Lie-group-based neural network algorithm.

The number of iterations and the loss function 𝓛(ξ,θ) are shown in Figure 7, where we observe that in this case, $𝓛\left(\theta \right)=5.4765\times 10^{-7}$, indicating the high accuracy of our approach.

**Figure 8:**
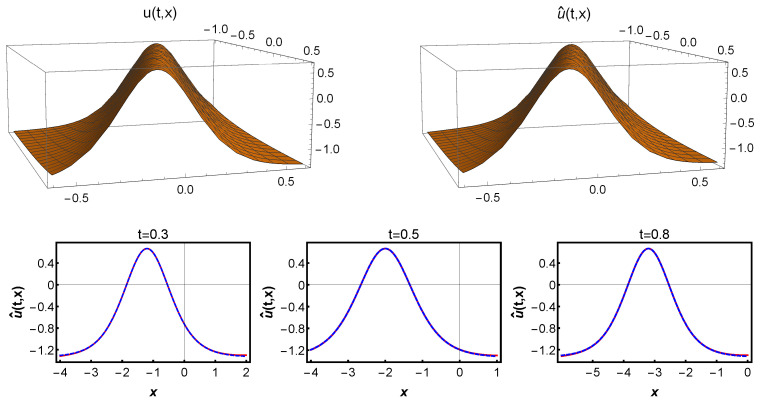
(**Top**): The true solution $u=2/3-2\tanh^{2}\left(x+4t\right)$ of the KdV equation is on the left, the predicted solution $\hat{u}\left(t,x\right)$ is on the right. (**Bottom**): Comparison of predicted and exact solutions at time t=0.3, 0.5, and 0.8. (The dashed blue line indicates the exact solution u(t,x), and the solid red line indicates the predicted solution $\hat{u}\left(t,x\right)$).

In Figure 8, we present a comparison between the true solution $u=2/3-2\tanh^{2}\left(x+4t\right)$ of the KdV equation (top left panel) and the predicted solution $\hat{u}$ (top right panel). Interestingly, the waveform of the single soliton does not change with time, as shown in the bottom panel of Figure 8 which gives the exact and predicted solutions for different times t=0.3, 0.5, and 0.8.

The complex nonlinear behavior of the KdV equation can be precisely captured by the Lie-groups-based neural network algorithm using just a minimal quantity of initial data (30 neurons in a single hidden layer with 250 training points).

## 4. Discussion and Conclusions

Our study focuses on the restoration of the dynamic behavior of the KdV equation using a Lie-group-based neural network algorithm. Compare with the existing PINN learning method, experimental findings demonstrate that our proposed method can accurately restore the dynamic behavior of the KdV equation with high accuracy and fast convergence under a small number of parameters and a simple network structure. In addition, a deep study is done for our proposed algorithm, and the accuracy is improved when the number of hidden layers increases with the number of neurons contained, but the time cost spent is also relatively high.

To confirm the accuracy and reliability of our proposed algorithm, we searched for other solitary solutions of the KdV equation. In the study presented in [31], an evaluation related to the design and efficacy of automatic tools for the derivation of solitary solutions of nonlinear differential equations is discussed. The study confirms by proof that the technique fails when considering the space of system parameters and initial conditions. To overcome these challenges, we can learn existing learning methods, such as the PINN method, to add both the errors generated by the initial and boundary conditions into the loss function. The change in our proposed algorithm in the way the loss function is constructed is made $L=L_{I}+L_{F}$, where $L_{I}$ is the error generated by the network solution $\hat{u}$ in the initial or boundary term. This modification to the construction of the loss function ensures that errors arising from both the initial and boundary conditions are considered in the prediction process, leading to more accurate results overall. Furthermore, the choice of operator $D_{1}$ plays a crucial role in subsequent neural network computation, and selecting the appropriate operator is vital to ensure precise and reliable results.

Notably, the success of our approach depends on capturing the mathematical substance of the equation solutions, which is often overlooked in machine learning techniques used for numerical solutions of differential equations. Recent research has shown that more implicit information about the solutions could be ignored when using these approaches. However, our proposed algorithm overcomes this limitation with only a shallow neural network model and limited data. Through our validation process, we have shown that our proposed algorithm performs effectively, producing highly accurate predictions for solitary solutions of the KdV equation. Our approach offers a new avenue for accurately predicting complex nonlinear solutions of PDEs and lays the foundation for future studies into other similar problems, which motivates us to study models in other interdisciplinary fields, such as finance or medical biology. The future work requires more research on optimization techniques to improve performance in addition to addressing parameter constraints or initial value constraints encountered in appeal problems.

## Figures and Tables

**Table 1:** The loss function 𝓛(θ) for different number of hidden layers and different number of neurons per layer. Here, the training points are fixed to the range [−3,3] of 250 uniformly spaced points.

Neurons			
𝓛(θ)	30	40	50
Layers			
1	2.3×10−7	1.9×10−7	2.0×10−7
2	3.1×10−8	3.5×10−8	2.7×10−8
3	1.6×10−8	1.8×10−8	1.5×10−8

## Data Availability

The data used to support the findings of this study are included within the article. The link to the code is https://github.com/yingWWen/Learning_the_nonlinear_solitary_wave_solution_of_KdV_equation_with_novel_neural_network_algorithm, accessed on 16 March 2023.
